# Injection of Adipose-Derived Stromal Vascular Fraction Rapidly Relieves Pain in Patients with Knee Osteoarthritis

**DOI:** 10.3390/medicina62020409

**Published:** 2026-02-20

**Authors:** Yong Sang Kim, Dong Suk Suh, Yoo Beom Kwon, Jai Hyun Chung, Yong Gon Koh

**Affiliations:** Center for Stem Cell & Arthritis Research, Department of Orthopaedic Surgery, Yonsei Sarang Hospital, 06073 Seoul, Republic of Korea; yskimos@gmail.com (Y.S.K.); lawnlei@naver.com (D.S.S.); run2kevk@gmail.com (Y.B.K.); jjhsky0843@gmail.com (J.H.C.)

**Keywords:** knee osteoarthritis, stromal vascular fraction, intra-articular injection, pain relief, dose–response relationship

## Abstract

*Background and Objectives*: Intra-articular injection of adipose-derived stromal vascular fraction (SVF) has emerged as a promising regenerative treatment for knee osteoarthritis (OA) because of its heterogeneous cellular composition and potent anti-inflammatory paracrine effects. Although SVF therapy has demonstrated clinical efficacy, the timing of pain relief and the influence of SVF cell dose on early clinical outcomes remain incompletely defined. *Materials and Methods*: This retrospective study included 146 patients (217 knees) with Kellgren–Lawrence (K–L) grade II–IV knee OA who underwent intra-articular injection of autologous adipose-derived SVF and completed a minimum follow-up of 1 year. Pain was assessed using the visual analog scale (VAS), and patients reported the time to perceived pain improvement after treatment. Radiographic severity was evaluated using the K–L grading system. Correlation analyses were performed to assess associations between pain-related outcomes, SVF cell number, and radiographic severity. *Results*: VAS scores improved significantly from baseline to the final follow-up (*p* < 0.01). Patients reported perceived pain improvement at a mean of 18.9 ± 14.5 days after SVF injection. The mean injected dose was 7.4 × 10^7^ total SVF cells per knee, including approximately 7.0 × 10^6^ stromal cells. Higher SVF cell numbers were significantly associated with greater pain improvement and lower VAS scores at final follow-up (*p* < 0.001 for both). Radiographic severity was not significantly correlated with pain at final follow-up, the magnitude of pain improvement, or the time to symptom relief. No clinically relevant adverse events were observed. *Conclusions*: Intra-articular injection of high-dose autologous SVF was associated with rapid and clinically meaningful pain relief, with symptom improvement occurring within approximately 3 weeks after treatment. The dose-dependent association and the lack of correlation with radiographic severity suggest that early pain relief is primarily mediated by the anti-inflammatory and paracrine effects of SVF rather than immediate structural cartilage regeneration.

## 1. Introduction

Knee osteoarthritis (OA) is one of the most prevalent degenerative joint diseases and a leading cause of chronic pain, disability, and reduced quality of life worldwide, with its prevalence steadily increasing due to population aging and rising obesity rates [1,2,3]. Knee OA is now recognized as a whole-joint disorder involving not only articular cartilage degeneration but also synovial inflammation, subchondral bone remodeling, and osteophyte formation, all of which contribute to persistent pain and progressive functional decline [1,2,4].

Conventional nonoperative treatments for knee OA, including non-steroidal anti-inflammatory drugs, physical therapy, and intra-articular injections of corticosteroids or hyaluronic acid, primarily provide symptomatic relief and fail to modify the underlying disease process [2,3]. Although total knee arthroplasty remains an effective treatment for end-stage OA, it is invasive, associated with perioperative risks, and unsuitable for many patients due to age, comorbidities, or personal preference [3]. These limitations have driven increasing interest in minimally invasive regenerative therapies capable of alleviating pain while potentially modulating the intra-articular disease environment in patients with knee OA [2,3].

Among emerging regenerative approaches, adipose tissue-derived cell therapies have attracted growing attention owing to the abundance, accessibility, and high cellular yield of adipose tissue compared with other mesenchymal stem cell (MSC) sources [3,5]. Stromal vascular fraction (SVF) is a heterogeneous, non-cultured cell population derived from adipose tissue and composed of adipose-derived stromal cells, endothelial progenitor cells, pericytes, fibroblasts, macrophages, lymphocytes, and various growth factors and cytokines [1,6,7]. Unlike cultured adipose-derived stromal cells, SVF can be harvested and administered in a single procedure without cell expansion, reducing processing time, cost, and regulatory complexity and making it particularly suitable for real-world clinical practice [8,9]. Preclinical and translational studies suggest that the therapeutic effects of SVF are mediated predominantly through paracrine and immunomodulatory mechanisms rather than direct cell engraftment or differentiation, including suppression of pro-inflammatory cytokines, enhancement of anabolic signaling, and polarization of macrophages toward an anti-inflammatory M2 phenotype [6,7]. Stromal vascular fraction (SVF) is a heterogeneous, non-cultured cell population composed of adipose-derived stromal cells, endothelial progenitor cells, pericytes, macrophages, and various immune cells. Accumulating experimental and translational evidence suggests that the therapeutic effects of SVF are mediated predominantly through paracrine and immunomodulatory mechanisms rather than direct cell engraftment or differentiation. These mechanisms include suppression of pro-inflammatory cytokines, enhancement of anti-inflammatory signaling, and modulation of the intra-articular microenvironment. In particular, SVF has been shown to promote macrophage polarization toward an anti-inflammatory M2 phenotype and to increase the secretion of cytokines such as interleukin-10, interleukin-1 receptor antagonist, and transforming growth factor–β, while reducing levels of pro-inflammatory mediators including interleukin-1β and tumor necrosis factor–α [10,11].

Clinically, multiple prospective studies, randomized controlled trials, and systematic reviews have demonstrated that intra-articular SVF injection is generally safe and associated with significant improvements in pain and functional outcomes, as assessed by the visual analog scale (VAS), Western Ontario and McMaster Universities Osteoarthritis Index (WOMAC), Knee injury and Osteoarthritis Outcome Score (KOOS), and Lysholm score [1,3,12,13,14,15]. Importantly, several comparative and observational studies indicate that SVF may provide relatively rapid pain relief after intra-articular injection, with early reductions in VAS scores observed during the initial post-treatment period [8,13,14,15,16,17]. This early analgesic effect is thought to reflect the heterogeneous cellular composition of SVF and its immediate paracrine anti-inflammatory activity within the joint environment [6,7].

Despite these encouraging findings, existing studies are limited by small sample sizes, heterogeneous treatment protocols, and inconsistent evaluation of early pain trajectories, leaving the rapid analgesic effects of SVF incompletely characterized, particularly in large real-world clinical cohorts [3,18]. Therefore, further investigation using larger real-world populations is warranted to better define the magnitude and timing of pain relief following intra-articular SVF injection in patients with knee osteoarthritis [2,3].

Accordingly, the purpose of the present study was to evaluate the clinical effectiveness of intra-articular injection of autologous adipose-derived SVF in patients with knee osteoarthritis, with particular emphasis on the rapid relief of pain following treatment.

## 2. Materials and Methods

### 2.1. Study Design and Participants

This study was reviewed and approved by the Institutional Review Board of our hospital, and written informed consent was obtained from all participants. We retrospectively reviewed the medical records of 151 consecutive patients with knee OA who were treated with intra-articular injections of SVF at our clinic and had completed 1 year of follow-up between July 2024 and November 2024. The inclusion criteria were knee OA with Kellgren–Lawrence (K-L) grade [19] II, III or IV confirmed by clinical evaluation, radiography, and magnetic resonance imaging (MRI); and symptoms of unilateral knee joint pain and/or functional limitations despite a minimum of 3 months treatment with oral non-steroidal anti-inflammatory drugs. The exclusion criteria were a previous history of steroid injection within 1 year; comorbidities in hip or ankle joints; or hematological or cardiovascular disease(s), systemic infection(s), or immunosuppressive disorder(s). Patients who had knee instability, varus or valgus malalignment of the knee joint of ≥5°, metabolic arthritis, joint infections, or large meniscal tears were also excluded. Of the 151 qualified patients, 3 dropped out and 2 were lost during the follow-up. Therefore, a total of 146 patients (217 knees) were included, comprising 50 men and 96 women, with a mean age of 64.3 years (range, 43–86 years). The mean preoperative body mass index was 26.4 kg/m^2^ (range, 22.8–28.9), and the distribution of radiographic severity according to the Kellgren–Lawrence grading system is summarized in Table 1.

### 2.2. Isolation of SVF from Subcutaneous Adipose Tissue

**Figure 1:**
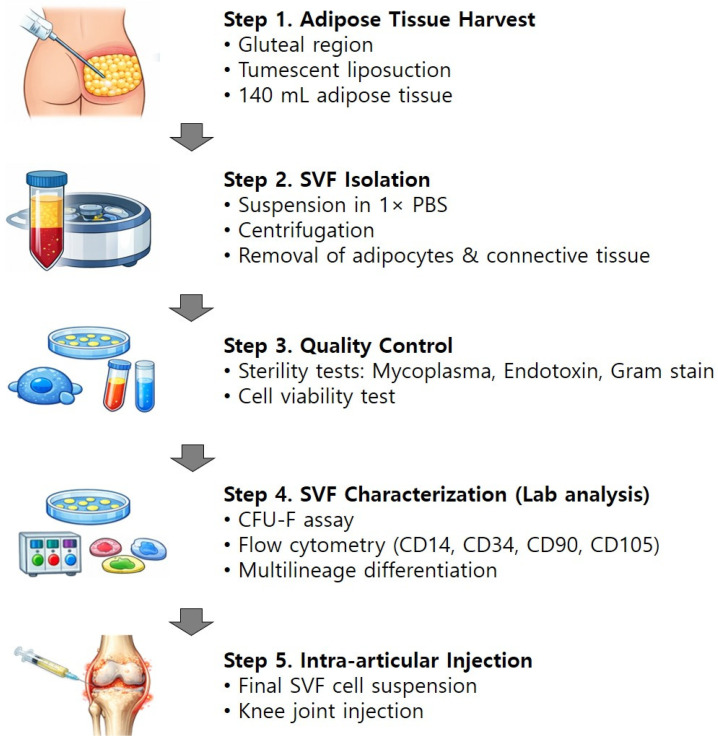
Schematic illustration of the stromal vascular fraction (SVF) process. Subcutaneous adipose tissue is harvested from the gluteal region by tumescent liposuction and processed to isolate SVF through suspension in phosphate-buffered saline and centrifugation. Quality control procedures, including sterility testing and cell viability assessment, are performed prior to clinical use. A portion of SVF is used for laboratory characterization, including colony-forming unit fibroblast (CFU-F) assays, flow cytometric immunophenotyping, and multilineage differentiation analyses. The final SVF cell suspension is prepared for intra-articular injection into the knee joint.

Subcutaneous adipose tissue samples were obtained through tumescent liposuction from the gluteal regions of patients 1 day before SVF injection. We collected 140 mL of adipose tissue, and this was suspended in phosphate-buffered saline (PBS) at the standard working concentration (1×), placed in a sterile box, and transported to the laboratory. A 120 mL of adipose tissue was used for injection and the remaining 20 mL of adipose tissue was processed similarly and used for laboratory analyses to characterize SVF cellular properties, including colony-forming unit fibroblast (CFU-F) formation and multilineage differentiation potential, as described in detail in the Supplementary Materials [20,21,22,23,24,25]. An overview of the SVF preparation and injection process is shown in Figure 1.

### 2.3. SVF Injection

All injections were performed using the same technique by an experienced senior orthopedic surgeon. Patients lay down on the table in a supine position with their knees extended during intra-articular injection. An arthrocentesis was performed to eliminate a knee effusion, before SVF was administered by transversely inserting a needle between the articular surface and patellofemoral joint in the midpoint of the patella, after pushing the patella upwards and shifting it to the lateral side (Figure 1) [26]. The patients were advised against additional treatments including physical therapy, acupuncture, steroid injection, and opioid or strong analgesics until 1 year after the injection. They were also told to avoid weight-bearing motions that impose an excessive burden on the affected knee, such as standing for prolonged periods, jogging, and lifting heavy objects, for the first 3 days.

### 2.4. Outcome Assessment

All patients were evaluated clinically and radiologically before injection and during follow-up(s). For clinical evaluation, the visual analog scale (VAS) for pain was collected and all patients were asked how soon after receiving injection treatment they began to feel improvement in pain. Adverse events were recorded for safety evaluation. Radiological evaluations included a weight-bearing anterior–posterior (AP) view, true lateral view at 30° of knee flexion, and hip-to-ankle standing AP radiograph on a long cassette. To avoid potential bias, an independent observer, who was a musculoskeletal-trained radiologist not involved in the care of patients and blinded to the intention of this study, performed the radiological evaluation. The K-L grading system [19] was used to assess the AP radiographs. Radiographic evaluations were performed at baseline prior to SVF injection and repeated at the final follow-up visit, approximately 12 months after treatment.

### 2.5. Statistical Analysis

The principal dependent variable was the VAS scores during the follow-up visits. Descriptive statistics were calculated as means ± standard deviations unless otherwise indicated. The Wilcoxon signed-rank test was used to evaluate differences between the preoperative and final follow-up values. Spearman’s rank-order correlation test was used to evaluate potential bivariate associations between different factors to identify significant correlations. Statistical analyses were performed using SPSS, Version 13.0 (IBM Corp., Armonk, NY, USA), and a *p*-value of <0.05 was considered statistically significant. Normality of continuous variables was assessed using the Shapiro–Wilk test. Because the data did not follow a normal distribution, non-parametric tests, including the Wilcoxon signed-rank test and Spearman’s rank-order correlation, were used.

## 3. Results

### 3.1. Pain Scores and Radiological Outcomes

The mean VAS score significantly improved from 6.5 ± 1.2 at baseline to 3.1 ± 1.6 at the final follow-up (*p* < 0.01). Patients reported a mean time to perceived pain improvement of 18.9 ± 14.5 days following SVF injection. No clinically significant adverse events were observed during the 12-month follow-up period. Although mild knee stiffness accompanied by swelling was reported in 9 patients, these symptoms resolved spontaneously without the need for additional intervention.

Radiographic evaluation using the Kellgren–Lawrence (K–L) grading system demonstrated that, prior to treatment, most knees were classified as grade II (94 knees, 43.3%), grade III (86 knees, 39.6%), or grade IV (37 knees, 17.1%) (Table 1). At the 1-year follow-up, the distribution of K–L grades remained largely unchanged, with grade II observed in 91 knees (41.9%), grade III in 87 knees (40.1%), and grade IV in 39 knees (18.0%), and no statistically significant difference was identified compared with baseline (*p* = 0.096).

### 3.2. Outcome Associations

Correlation analyses were performed to evaluate the associations between pain-related outcomes and SVF cell number, as well as radiographic disease severity assessed by the Kellgren–Lawrence (K–L) grading system (Table 2 and Table 3). No significant correlation was observed between SVF cell number and baseline VAS score (Spearman’s ρ = 0.088, *p* = 0.195). In contrast, SVF cell number demonstrated a significant negative correlation with VAS score at the final follow-up (ρ = −0.262, *p* < 0.001), indicating that a higher SVF cell number was associated with lower pain levels at 1 year after injection. Additionally, the magnitude of pain improvement, defined as the difference between baseline and final follow-up VAS scores, showed a moderate positive correlation with SVF cell number (ρ = 0.370, *p* < 0.001). A significant positive correlation was also identified between SVF cell number and the duration until symptom improvement (ρ = 0.219, *p* = 0.001), suggesting that patients receiving higher numbers of SVF cells tended to report symptom improvement over a longer time period (Table 2).

Associations between K–L grade and pain-related outcomes were generally weak (Table 3). Baseline K–L grade demonstrated a weak but statistically significant positive correlation with baseline VAS score (ρ = 0.151, *p* = 0.026), indicating higher baseline pain levels in patients with more advanced radiographic osteoarthritis. Similarly, final follow-up K–L grade was weakly correlated with baseline VAS score (ρ = 0.166, *p* = 0.014). However, neither baseline nor final follow-up K–L grade showed significant correlations with final follow-up VAS score, the degree of pain improvement, or the duration until symptom improvement (all *p* > 0.05), suggesting that radiographic severity was not strongly associated with post-treatment pain outcomes following SVF injection. The variability in pain response observed among patients may reflect differences in SVF cell composition and viability, injected cell dose, baseline inflammatory joint environment, and individual patient characteristics, underscoring the heterogeneous nature of osteoarthritis.

## 4. Discussion

The most notable finding of the present study is the rapid improvement in pain following intra-articular injection of autologous stromal vascular fraction (SVF), with patients reporting symptomatic relief at a mean of approximately 3 weeks after treatment. This early clinical response distinguishes our results from many previously published SVF studies and may be attributable, at least in part, to the relatively high SVF cell number administered in our cohort.

Although SVF therapy for knee osteoarthritis has been increasingly reported, most previous studies have focused on overall efficacy, safety, or comparative treatment strategies. In contrast, the present study specifically addressed three clinically relevant but underexplored aspects: the timing of pain relief, the relationship between SVF cell number and pain outcomes, and the influence of radiographic disease severity on clinical response. Our findings demonstrate that SVF injection results in rapid pain improvement, that greater pain relief is associated with higher SVF cell numbers, and that pain outcomes are not significantly influenced by Kellgren–Lawrence grade. These results provide practical insights into patient selection and treatment optimization beyond the general demonstration of efficacy reported in prior studies.

Previous clinical studies and systematic reviews have consistently demonstrated that intra-articular SVF injection is safe and effective for reducing pain and improving function in patients with knee osteoarthritis; however, the onset of symptom relief has typically been reported at follow-up intervals of 1 to 3 months rather than within the first few weeks [1,11]. Several trials and reviews have described meaningful improvements in pain scores beginning at 4–12 weeks after injection, highlighting substantial variability in the timing of clinical response across studies [11].

In a double-blind randomized self-controlled trial, Hong et al. [2]. reported that patients with Kellgren–Lawrence (K–L) grade II–III knee osteoarthritis experienced significant improvements in VAS and WOMAC scores beginning at 1 month after SVF injection, with continued improvement at subsequent follow-up visits. Notably, the SVF preparations used in that study generally yielded approximately 1–3 × 10^7^ total SVF cells per knee, which is substantially lower than the SVF cell number administered in the present study. Similarly, Lapuente et al. [10]. demonstrated significant clinical improvement at 1-year follow-up, with pain relief occurring progressively over the first several postoperative months rather than within weeks after injection. These findings suggest that, in most prior studies, early pain improvement within the first few weeks was not a predominant feature of SVF therapy.

Systematic reviews further support these observations. Shanmugasundaram et al. [11]. analyzed 11 clinical studies of SVF therapy and reported that the earliest consistent improvements in pain were observed between 4 and 12 weeks after treatment, with most studies administering SVF cell numbers in the range of 10^6^ to low 10^7^ cells. Likewise, Goncharov et al. [1]. highlighted the substantial heterogeneity in SVF preparation protocols and cell yields, noting that few studies directly examined the relationship between injected SVF cell number and the timing of pain relief, with most clinical improvements reported at 1–3 months after injection. A comparison of SVF cell dose and the timing of pain improvement across previously published studies and the present cohort is summarized in Table 4.

In contrast to these prior reports, the present study employed a higher mean SVF cell dose and demonstrated a significantly shorter time to perceived pain improvement. Correlation analysis further revealed that higher SVF cell numbers were significantly associated with greater reductions in VAS scores, supporting a potential dose-dependent relationship between SVF cell number and pain relief.

Importantly, radiographic disease severity assessed using the K–L grading system was not significantly associated with pain outcomes, including pain at final follow-up, the magnitude of pain improvement, or the time to perceived symptom relief. This finding suggests that the observed pain reduction following SVF injection may occur independently of structural disease severity, as reflected by radiographic grading. Several previous studies have similarly reported a discordance between radiographic severity and clinical pain outcomes following SVF therapy, indicating that pain relief may precede or occur independently of structural cartilage repair [2,11].

From a mechanistic perspective, the rapid pain relief observed in the present study may be explained by the paracrine and immunomodulatory effects of SVF rather than by immediate structural cartilage regeneration. The heterogeneous cellular composition of SVF allows for early modulation of the inflammatory joint environment, which may contribute to prompt symptom improvement. This interpretation is supported by previous clinical and biochemical studies demonstrating that SVF injection leads to rapid reductions in pro-inflammatory cytokines and increases in anti-inflammatory mediators within the synovial fluid, occurring in parallel with clinical pain relief [10,11].

Lapuente et al. [10]. further provided clinical and biochemical evidence supporting this mechanism by demonstrating significant reductions in pro-inflammatory cytokines and matrix metalloproteinases within the synovial fluid following SVF injection, accompanied by increases in anabolic and anti-inflammatory mediators. Importantly, these molecular changes occurred in parallel with clinical pain improvement, reinforcing the concept that symptom relief is driven primarily by immunomodulation rather than immediate cartilage regeneration.

Clinical comparative studies further corroborate the predominance of paracrine effects in SVF therapy. Yokota et al. [9]. reported that although both SVF and cultured adipose-derived stromal cell injections significantly improved pain and function, SVF-treated knees exhibited earlier symptomatic improvement, whereas stromal cell therapy demonstrated more sustained long-term structural and functional benefits. Similarly, a recent systematic review focusing on K–L grade II–III osteoarthritis concluded that SVF therapy is characterized by rapid paracrine-driven pain reduction, while cartilage regeneration, when present, likely contributes to longer-term outcomes rather than early symptom improvement [3].

From a clinical standpoint, the ability to achieve early pain relief is highly relevant. Rapid symptom improvement may enhance patient satisfaction, reduce reliance on analgesic medications, and facilitate earlier engagement in rehabilitation and daily activities. The early analgesic effect observed across a range of K–L grades in the present study suggests that SVF therapy with an adequate cell dose may be broadly applicable in patients with knee osteoarthritis.

This study has several limitations. First, its retrospective design and the absence of a control group limit the ability to draw definitive causal conclusions regarding the observed clinical improvements. The absence of a control group receiving conventional therapy represents a major limitation of this study. However, our primary objective was to evaluate the early pain trajectory and dose–response relationship of SVF therapy in a large real-world cohort, rather than to compare its efficacy with established treatments, which has already been addressed in previous randomized controlled trials and systematic reviews. Future prospective randomized studies incorporating conventional therapy control groups and combination treatment arms are warranted to further clarify the comparative and synergistic effects of SVF therapy.

Second, although multidimensional outcome measures such as the WOMAC pain score provide comprehensive assessment of osteoarthritis-related symptoms, the use of VAS allowed consistent evaluation of pain intensity and early symptom changes across the entire cohort, which aligned with the primary objective of assessing the timing of pain relief following SVF injection. Third, although a significant association between SVF cell number and pain reduction was identified, the optimal SVF cell dose required to maximize both early and long-term clinical benefits remains undefined. Fourth, radiographic outcomes were assessed using conventional grading systems, which may not fully capture subtle structural changes or biological remodeling within the joint. Radiographic progression of knee osteoarthritis is typically slow, and meaningful changes in Kellgren–Lawrence grade are unlikely to be detected within a one-year period. Therefore, the absence of radiographic progression data in this study limits conclusions regarding disease-modifying effects of SVF therapy. Despite the lack of structural outcome measures, the rapid and sustained pain relief observed in this cohort suggests that SVF therapy may exert symptomatic benefits that precede detectable structural changes. Finally, heterogeneity in patient characteristics and SVF preparation techniques may have influenced treatment response. Future prospective, randomized, dose-comparison studies with standardized SVF processing protocols and longer follow-up are warranted to address these limitations.

## 5. Conclusions

In conclusion, intra-articular injection of autologous SVF resulted in rapid and clinically meaningful pain relief, with symptom improvement occurring within approximately 3 weeks after treatment. The lack of association between radiographic disease severity and pain outcomes, together with evidence from prior clinical and experimental studies, suggests that this early analgesic effect is predominantly mediated by the anti-inflammatory and paracrine actions of SVF, rather than by immediate structural cartilage regeneration. These findings highlight the potential of high-dose SVF therapy as an effective, biologically active, and broadly applicable treatment option for patients with knee osteoarthritis, particularly for achieving early symptomatic benefit.

## Figures and Tables

**Table 1:** Baseline characteristics.

Age, y	64.3 ± 7.2 (43–86)
Sex, male/female, *n*	50/96
Side of involvement, right/left, *n*	107/110
Body mass index, kg/m^2^	26.4 ± 1.6 (22.8–28.9)
Kellgren–Lawrence grade, *n* (%)	
II	94 (43.3)
III	86 (39.6)
IV	37 (17.1)

Data are presented as means ± standard deviation (range) unless otherwise indicated.

**Table 2:** Correlations between Pain Outcomes and SVF Number ^a^.

	SVF Number	
	S rho	*p* Value
Initial VAS	0.088	n.s.
Final follow-up VAS	−0.262	<0.001
Difference between initial and final follow-up VAS	0.370	<0.001
Duration until symptom improvement, days	0.219	n.s.

^a^ Calculated using the Spearman rank-order test. VAS, visual analog scale; S, Spearman; SVF, stromal vascular fraction.

**Table 3:** Correlations between Pain Outcomes and K-L Grade at Baseline and at the Final Follow-up ^a^.

	K-L Grade			
	Baseline		Final Follow-Up	
	S rho	*p* Value	S rho	*p* Value
Initial VAS	0.151	n.s.	0.166	n.s.
Final follow-up VAS	0.125	n.s.	0.120	n.s.
Difference between initial and final follow-up VAS	−0.010	n.s.	0.001	n.s.
Duration until symptom improvement, days	−0.057	n.s.	−0.056	n.s.

^a^ Calculated using the Spearman rank-order test. VAS, visual analog scale; S, Spearman; K-L, Kellgren–Lawrence.

**Table 4:** Comparison of SVF Cell Number and Time to Pain Improvement Across Studies ^a^.

Study	Study Design/OA Grade	Reported SVF Cell Number (per Knee)	Reported Time to Pain Improvement	Key Notes
Hong et al., 2019 [2]	Double-blind randomized self-controlled trial/K–L II–III	≈1–3 × 10^7^ total SVF cells	From 1 month	VAS and WOMAC improvement reported at 1, 3, 6, and 12 months
Lapuente et al., 2020 [10]	Retrospective cohort/moderate–severe OA	Lower SVF yield; exact number not standardized	Several months; primary endpoint at 1 year	Focus on immunomodulatory and long-term effects
Shanmugasundaram et al., 2021 (Systematic review) [11]	11 clinical studies/mixed OA grades	Typically 10^6^ to low 10^7^ cells	4–12 weeks	Marked heterogeneity in SVF preparation
Goncharov et al., 2023 (Systematic review) [1]	22 studies/knee OA	Mostly within 10^6^–10^7^ cells	1–3 months	Dose–response relationship rarely analyzed
Yokota et al., 2022 [9]	Parallel single-arm trials/K–L II–IV	SVF dose lower than cultured ASC protocols	Earlier than ASCs, but not within weeks	ASC superior for long-term outcomes
Present study	Retrospective cohort/K–L II–IV	7.4 × 10^7^ total SVF cells (≈7.0 × 10^6^ stromal cells)	18.9 ± 14.5 days (≈3 weeks)	Shortest time to pain improvement among compared studies

^a^ SVF, stromal vascular fraction; ASC, adipose-derived stromal cell; OA, osteoarthritis; K–L, Kellgren–Lawrence; VAS, visual analog scale; WOMAC, Western Ontario and McMaster Universities Osteoarthritis Index.

## Data Availability

The data presented in this study are available on request from the corresponding author.

## Supplementary Materials

The following supporting information can be downloaded at: https://www.mdpi.com/article/10.3390/medicina62020409/s1.
