# Supplementary material for: Injection of Adipose-Derived Stromal Vascular Fraction Rapidly Relieves Pain in Patients with Knee Osteoarthritis
Source: Medicina (Kaunas). 2026 Feb 20;62(2):409. doi: 10.3390/medicina62020409 (PMC12943560; doi:10.3390/medicina62020409)
Supplement: Supplementary file 1 [file medicina-62-00409-s001.zip › medicina-4118884-supplementary.pdf]

Supplementary Material

Methods

Isolation and Processing of Stromal Vascular Fraction (SVF)

Subcutaneous adipose tissue was harvested from the gluteal region using tumescent liposuction 1 day prior to intra-articular injection. A total of 140 mL of adipose tissue was collected and suspended in phosphate-buffered saline (PBS) at the standard working concentration (1×). The samples were placed in a sterile container and transported to the laboratory under controlled conditions.

Mature adipocytes and connective tissue components were separated from the stromal vascular fraction (SVF) by centrifugation (Hanil Scientific Inc., Gyeonggi-do, South Korea), following previously described protocols [1]. Of the collected adipose tissue, 120 mL was allocated for clinical injection. Prior to administration, sterility testing was performed, including mycoplasma testing (iNtRON, Gyeonggi-do, South Korea), endotoxin assays (Associates of Cape Cod, MA, USA), and Gram staining (BD Biociences, Franklin Lakes, NJ, USA). Cell viability was assessed using the methylene blue dye exclusion test (NanoEntek, Seoul, South Korea).

The remaining 20 mL of adipose tissue was processed in the same manner and used for laboratory analyses to characterize the cellular properties of SVF, including colony-forming unit fibroblast (CFU-F) formation and multilineage differentiation capacity.

Colony-Forming Unit Fibroblast (CFU-F) Assay

To confirm the presence of mesenchymal progenitor cells, a CFU-F assay was performed. Cells

derived from SVF were plated at low density (16 cells/cm²) in T25 culture flasks and maintained under standard culture conditions. After incubation, colonies consisting of aggregates of at least 50 cells were identified and counted under an optical microscope to evaluate colony-forming ability [2,3].

Flow Cytometric Immunophenotyping

Cells were expanded in culture at a seeding density of 50 cells/cm² to obtain sufficient cell numbers for immunophenotypic analysis. Flow cytometric analysis was conducted using fluorescence-activated cell sorting (FACS) to assess the expression of mesenchymal and hematopoietic surface markers. Antibodies against CD14, CD34, CD90, and CD105 were used according to established protocols [4,5]. For each marker, 2×10^6 cells were required; therefore, a total of 8×10^6 cells were obtained through culture expansion for analysis of all four markers.

Multilineage Differentiation Assays

To evaluate multilineage differentiation potential, adipose-derived stromal cells were plated at a density of 5×10^3 cells/cm² in Dulbecco's modified Eagle's medium (DMEM; HyClone, Logan, UT, USA) supplemented with 10% fetal bovine serum (FBS; HyClone). After 24 hours of adherence, the culture medium was replaced with lineage-specific inductive media to assess adipogenic, osteogenic, and chondrogenic differentiation, as previously described [6]. Differentiation was confirmed using standard histological staining methods specific to each lineage.

Results

CFU-F Formation

The CFU-F assay demonstrated the presence of plastic-adherent fibroblast-like colonies derived from SVF cells, confirming the existence of mesenchymal progenitor cells within the adipose-derived cell population.

Cellular Composition of SVF

Quantitative analysis revealed that adipose-derived stromal cells comprised approximately 9.6% of the total SVF cell population. The SVF preparations contained an average of 7.0×10^6 stromal cells and an average total SVF cell count of 7.3×10^7 cells per sample (range, 6.5–8.4 $\times 10^7$ cells), which were used for clinical implantation.

Flow Cytometric Immunophenotype

Flow cytometric analysis demonstrated high expression of mesenchymal stem cell markers CD90 (99.14%) and CD105 (93.73%), with low expression of hematopoietic and myeloid markers CD34 (5.17%) and CD14 (2.46%), consistent with the immunophenotypic profile of mesenchymal stromal cells.

Multilineage Differentiation Potential

Cells derived from SVF exhibited adipogenic, osteogenic, and chondrogenic differentiation potential, as confirmed by lineage-specific staining assays, supporting the multipotent characteristics of adipose-derived stromal cells.

References

1. Zuk, P.A.; Zhu, M.; Mizuno, H.; Huang, J.; Futrell, J.W.; Katz, A.J.; Benhaim, P.; Lorenz, H.P.; Hedrick, M.H. Multilineage cells from human adipose tissue: implications for cell-based therapies. *Tissue Eng* **2001**, *7*, 211-228; DOI:10.1089/107632701300062859.
2. Prockop, D.J. Marrow stromal cells as stem cells for nonhematopoietic tissues. *Science* **1997**, *276*, 71-74; DOI:10.1126/science.276.5309.71.
3. Friedenstein, A.J.; Chailakhyan, R.K.; Latsinik, N.V.; Panasyuk, A.F.; Keiliss-Borok, I.V. Stromal cells responsible for transferring the microenvironment of the hemopoietic tissues. Cloning in vitro and retransplantation in vivo. *Transplantation* **1974**, *17*, 331-340; DOI:10.1097/00007890-197404000-00001.
4. Smith, M.B.; Whiteside, M.G.; Campbell, D.G. The occurrence of heterozygous beta-thalassaemia as screened by quantitative haemoglobin electrophoresis in pregnancy. *Med J Aust* **1971**, *1*, 1273-1274; DOI:10.5694/j.1326-5377.1971.tb92389.x.
5. de Girolamo, L.; Niada, S.; Arrigoni, E.; Di Giancamillo, A.; Domeneghini, C.; Dadsetan, M.; Yaszemski, M.J.; Gastaldi, D.; Vena, P.; Taffetani, M. *et al.* Repair of osteochondral defects in the minipig model by OPF hydrogel loaded with adipose-derived mesenchymal stem cells. *Regen Med* **2015**, *10*, 135-151; DOI:10.2217/rme.14.77.
6. Marchal, J.A.; Picón, M.; Perán, M.; Bueno, C.; Jiménez-Navarro, M.; Carrillo, E.; Boulaiz, H.; Rodríguez, N.; Álvarez, P.; Menendez, P. *et al.* Purification and long-term expansion of multipotent endothelial-like cells with potential cardiovascular regeneration. *Stem Cells Dev* **2012**, *21*, 562-574; DOI:10.1089/scd.2011.0072.
